# Brain-wide functional connectivity of face patch neurons during rest

**DOI:** 10.1073/pnas.2206559119

**Published:** 2022-08-31

**Authors:** Daniel Zaldivar, Kenji W. Koyano, Frank Q. Ye, David C. Godlove, Soo Hyun Park, Brian E. Russ, Rebecca Bhik-Ghanie, David A. Leopold

**Affiliations:** ^a^Section on Cognitive Neurophysiology and Imaging, National Institute of Mental Health, National Institutes of Health, Bethesda, MD 20892;; ^b^Neurophysiology Imaging Facility, National Institute of Mental Health, National Institute for Neurological Disorders and Stroke, National Eye Institute, National Institutes of Health, Bethesda, MD 20892

**Keywords:** simultaneous fMRI and neurophysiology, single units, resting state, face patches, brain networks

## Abstract

The brain is a highly organized, dynamic system whose network architecture is often assessed through resting functional magnetic resonance imaging (fMRI) functional connectivity. The functional interactions between brain areas, including those observed during rest, are assumed to stem from the collective influence of action potentials carried by long-range neural projections. However, the contribution of individual neurons to brain-wide functional connectivity has not been systematically assessed. Here we developed a method to concurrently measure and compare the spiking activity of local neurons with fMRI signals measured across the brain during rest. We recorded spontaneous activity from neural populations in cortical face patches in the macaque during fMRI scanning sessions. Individual cells exhibited prominent, bilateral coupling with fMRI fluctuations in a restricted set of cortical areas inside and outside the face patch network, partially matching the pattern of known anatomical projections. Within each face patch population, a subset of neurons was positively coupled with the face patch network and another was negatively coupled. The same cells showed inverse correlations with distinct subcortical structures, most notably the lateral geniculate nucleus and brainstem neuromodulatory centers. Corresponding connectivity maps derived from fMRI seeds and local field potentials differed from the single unit maps, particularly in subcortical areas. Together, the results demonstrate that the spiking fluctuations of neurons are selectively coupled with discrete brain regions, with the coupling governed in part by anatomical network connections and in part by indirect neuromodulatory pathways.

## Significance

In resting state functional magnetic resonance imaging (fMRI), areas showing coherent hemodynamic fluctuations across the brain are operationally defined to be functionally connected. However, it is unknown how the activity of single units residing within a voxel contributes to this network structure. Here we demonstrate a shared but restricted pattern of functional connectivity among neighboring neurons residing in functionally defined face patches. Unexpectedly, such neurons also exhibited a prominent inverse correlation with thalamic structures and brainstem neuromodulatory centers. Single unit maps differed from analogous maps obtained with local field potentials and seed-based fMRI. These findings suggest that during rest, individual cortical neurons have a restricted set of functional connections, which is governed in part by anatomical projections and in part by neuromodulation.

Normal brain function entails a complex interplay among neural networks operating at multiple spatial and temporal scales (1, 2). In the last decades, functional magnetic resonance imaging (fMRI) has enabled researchers to noninvasively study the structure and organization of these neural networks through resting state fMRI (rs-fMRI). This analysis uses the covariation of spontaneous fluctuations across the brain to identify functionally connected regions (3, 4) that together constitute a functional network (5, 6). Importantly, the networks revealed by this analysis often resemble those derived from task-based studies, such as those involving motor action (6), cognitive operation (7), or perceptual experience (8).

Despite the widespread use of fMRI-based functional connectivity in humans, its neural basis has not been studied systematically in animals, where local neural circuit activity can be assessed via invasive methodologies. The absence of research in this area can be understood, in part, by the significant challenges associated with concurrently measuring neural and fMRI signals. In the macaque, a few studies have used simultaneous measurement of neural and fMRI signals to study spontaneous activity, for example, comparing spontaneous field potential fluctuations to regional fMRI activity (9) or to global fMRI fluctuations (10). One experiment went further by mapping the brain-wide coupling of spontaneous field potential events linked to particular cognitive operations (11). These studies, though inherently limited by the different spatiotemporal scales of neural and hemodynamic signals (12), have been valuable for assessing how local neural information is coupled with activity elsewhere in the brain.

The contribution of individual neurons in functional connectivity is unclear. The observed spatiotemporal correlations are probably constrained by the pattern of axonal connections, which permit the direct exchange of signals across remote brain areas. However, the relationship between functional connectivity and anatomical connections is imprecise (13, 14). Moreover, the functional connectivity of individual neurons, such as those populating an rs-fMRI voxel, has not been studied. Cortical neurons, like fMRI voxels, exhibit large-amplitude, slow fluctuations in their spiking over time scales of many seconds (15). Do these spiking fluctuations underlie the spatiotemporal correlations measured with rs-fMRI? If so, how would the functional connectivity computed from a given neuron compare to that of a neighboring neuron, to the local field potential (LFP), and to locally measured fMRI signal obtained from the same tissue? Ideally, these questions would be best addressed in the context of an fMRI network whose neurophysiology and anatomy are well studied.

The face patch system in the macaque is a network of regions in the inferior temporal cortex defined by its selective visual responses to faces (16, 17). These face patches are composed of circumscribed clusters of cells that have been implicated in the structural analysis of faces (18–21). Previous work has investigated the anatomical (22, 23) and fMRI functional (8) connectivity of face patches to demonstrate a highly interconnected network. Based on these results, one might expect that the neurons within individual nodes of this network would show activity correlations with a restricted set of areas and most strongly with other face patches. However, in some modes of visual stimulation this is not the case. For example, during the viewing of naturalistic movies, face patch neurons exhibited varied patterns of correlated activity with visually driven networks, with neighboring neurons often yielding highly distinct correlation maps (24, 25). These results prompt the question of whether neurons in a face patch population would show a restricted and homogeneous pattern of functional connectivity across the brain at rest or whether they would exhibit a high level of diversity in their brain-wide functional connectivity.

In the present study, we investigated the resting state functional connectivity of individual neurons by recording from local neural populations in the anterior fundus (AF) and anterior medial (AM) face patches. Concurrent single unit recordings and fMRI were carried out in darkness inside the scanner bore, with animals at rest and not performing any task. We created brain-wide correlation maps by comparing ongoing spiking fluctuations of isolated neurons with fMRI time courses across the brain, in a method akin to seed-based fMRI functional connectivity. The results revealed prominent fMRI coupling across a restricted set of cortical areas, with the strongest correlations observed with voxels in the face patch network, V4, TEO, and the ventral premotor cortex. Neurons within each local population and across the two face patches exhibited a high level of overlap in their pattern of functional connectivity. Unexpectedly, the same neurons showed a strong inverse correlation with the lateral geniculate nucleus (LGN) and brainstem neuromodulatory centers, a feature that was absent in corresponding maps computed from the LFP or fMRI seed voxel derived from the same recording locations.

## Results

**Fig. 1.**
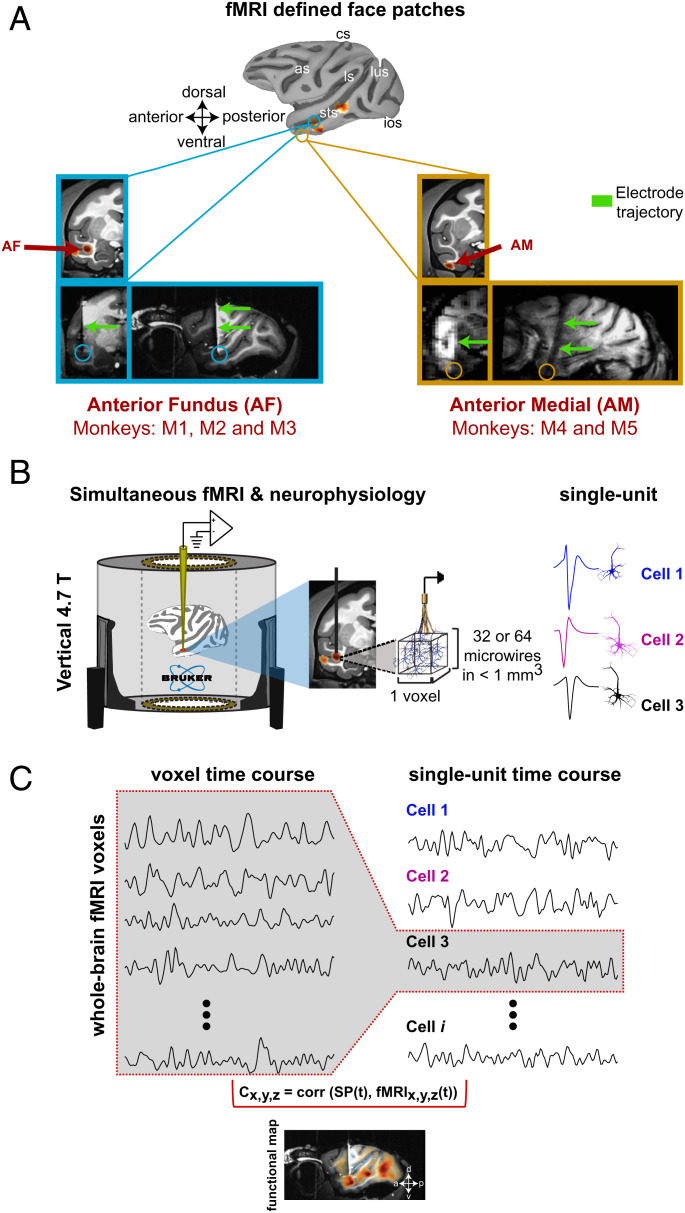
Single unit fMRI experiments in the resting macaque. (*A*) Location of the fMRI-defined face patches from which single units were recorded. *Top*: macaque cortical surface depicting the location of face selective areas (see also *SI Appendix*, Fig. S1 for an example of the block design stimulus presentation): as, arcuate sulcus; cs, central sulcus; ios, inferior occipital sulcus; ls, lateral sulcus; lus, lunate sulcus; sts, superior temporal sulcus*.* Structural MRI and functional overlay showing activation in voxels corresponding to the face patch AF (anterior fundus in monkey M1; blue box) and AM (anterior medial in monkey M4; yellow box). Coronal (*Left*) and sagittal (*Right*) T1-weighted anatomical images depicting the electrode location corresponding to AM (yellow box) and AF (blue box). Electrode trajectory is indicated by the green arrows. Below the animal identifications for each face patch recordings are listed. (*B*) fMRI and neurophysiology experiments were conducted simultaneously inside a 4.7-T vertical scanner wherein monkeys were resting and with no sensory stimulus. Neurophysiological recordings were carried out with bundles of either 32 or 64 MR-compatible microwires chronically implanted in a face patch. (*C*) Example of time courses from fMRI voxel activity (*Left*) and single unit spiking fluctuations (*Right*). We compared single unit time series with the voxel time courses throughout the brain. A representative example of single unit fMRI correlation map is shown on a sagittal section. Color encodes the Spearman’s rank correlation coefficient from each voxel across voxels in the whole brain.

We created fMRI correlation maps from single units based on concurrent electrophysiological and fMRI acquisition. For this, we chronically implanted magnetic resonance (MR)-compatible microwire electrode bundles (26) into the cerebral cortex of five nonhuman primates, permitting longitudinal and stable recordings from multiple neurons in the MRI scanner environment (20, 26). The neural recordings were performed from the AF and AM face patches (Fig. 1*A*), which are considered to be advanced processing stages within the face patch network (19). These areas were initially identified with standard fMRI face patch localizers in each monkey (*SI Appendix*, Fig. S1 *A*–*C*) (20). During the concurrent recordings, the animals sat quietly in the dark scanner without any task or visual stimulation. While their behavior was not tracked systematically, their eyes opened and closed during scanning, indicating arousal fluctuations with periods of light sleep, as described in a previous study using the same scanning protocol (27).

The electrophysiological signals were recorded with filtered cables and high-input-range amplifiers (PZ5 NeuroDigitizer Amplifier, Tucker Davis Technologies [TDT]) configured to record single unit activity inside the scanner bore during whole-brain fMRI scans (Fig. 1 and see *Materials and Methods*). The fMRI acquisition included 1.2-s gaps between successive volumes, during which time the electrophysiological signal was relatively unpolluted. Following the removal of radiofrequency and gradient artifacts from recording traces, analysis of spiking and field potential was restricted to these gap periods (see *Materials and Methods*) (10). In total, we recorded the activity of 157 isolated, distinct single units inside the MRI scanner. Of these neurons, 79 units were recorded from AF (36 in M1, 30 in M2 and, 13 in M3) and 78 units were recorded from AM (34 in M4 and 44 in M5). Brain-wide correlation maps were created by computing the Spearman coefficient for each voxel after convolving single-trial spike rate fluctuations with the hemodynamic response function and then down-sampling to match the repetition time (TR) of the fMRI acquisition (see *Materials and Methods*). As the microwires permitted longitudinal and stable tracking of individual neurons (28, 29), we combined data for single neurons across multiple sessions when evidence suggested the isolation had been maintained. To facilitate the comparison of the resulting functional activations across multiple animals and days, we aligned and projected the correlation maps onto the National Institute of Mental Health Macaque Template (NMT) (30).

### fMRI Coupling Profiles of Face Patch Neurons.

**Fig. 2.**
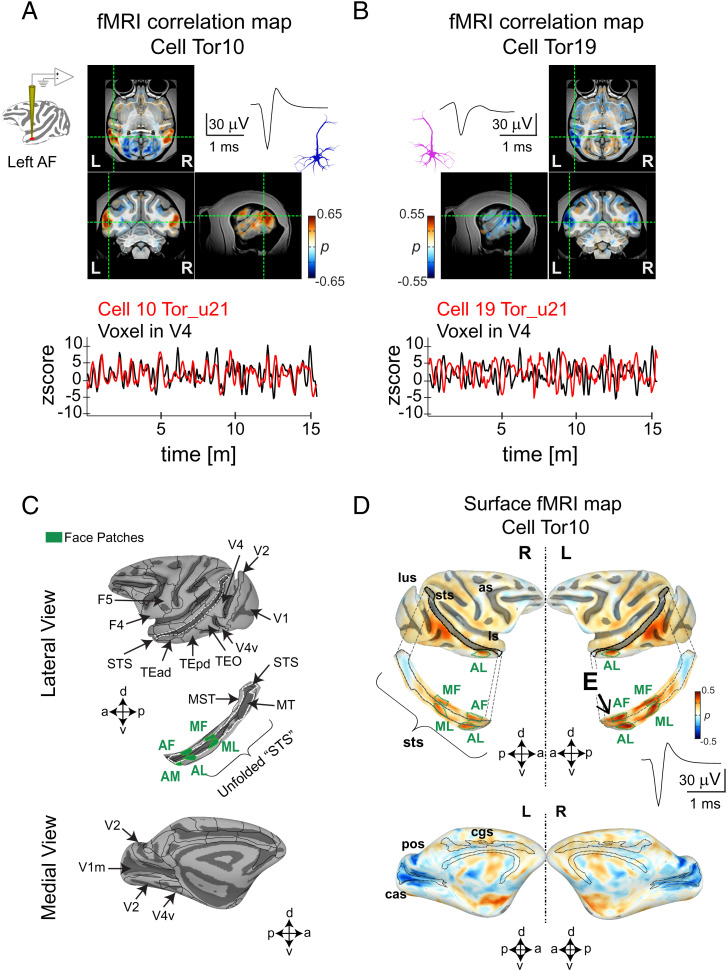
Examples of two single unit functional maps from neurons in the macaque AF face patch. fMRI maps (*Top Panel*) obtained from (*A*) cell Tor10 and (*B*) cell Tor19. The two example cells were recorded from the left hemisphere in monkey M1 during the same scan session (u21). The activation maps from these cells are displayed in the axial, coronal, and sagittal planes and are accompanied by their respective spike waves. Scale bar, 30 mV, 1 ms. Overlaid color maps represent the Spearman’s rank correlation coefficients for each voxel. Time series (*Bottom Panel*) corresponding to (*A*) cell Tor10 and to (*B*) cell Tor19 (both shown in red) are compared with the activity of an fMRI voxel time series (black). The location of the voxel time series is indicated by the dotted green lines on each plane (see also *SI Appendix*, Fig. S2 *A* and *B* for the whole brain functional maps corresponding to these cells). (*C*) Lateral (*Top Panel*) and medial (*Bottom Panel*) views of a macaque surface depicting the location of cortical regions relevant to the current study. Boundaries of functionally defined face patches are superimposed (green) in an unfolded STS. Abbreviations for face patches: AL, anterior lateral; MF, middle fundus; ML, middle lateral. (*D*), Averaged single unit functional map (*n* = 12 scans, 30 m each, total scan time 6 h), accompanied by the corresponding spike wave from cell Tor10 is plotted in the lateral (*Top*) and medial (*Bottom*) view of the cortical surface: as, arcuate sulcus; cas, calcarine sulcus; ios, inferior occipital sulcus; ls, lateral sulcus; lus, lunate sulcus; pos, parieto-occipital sulcus; cgs, cingulate sulcus; sts, superior temporal sulcus (see also *SI Appendix*, Fig. S2*C* for the functional maps stability across days). Activity in face patches is depicted in the unfolded STS surface where “E” indicates the electrode location.

Maps from two representative AF neurons, recorded from the same single session (session u21, monkey M1), are shown in Fig. 2 *A* and *B*. The neuron in the first example (cell Tor10, Fig. 2*A*) exhibited bilateral positive correlations in the cortex that were strongest in other face patches and areas V4 and TEO. As was representative of the neural population, the voxels showing the highest absolute correlations were remote from the neural recording location in AF. For this neuron, there was also a prominent negative correlation in the medial aspect of V1 (V1m). Beneath the maps is a comparison of the neuron’s spiking time course (red line, Fig. 2*A*) with that of a single fMRI voxel in V4 (black line, Fig. 2*A*), demonstrating a close correspondence over a 15-m recording period. The time course of a neighboring face patch neuron recorded simultaneously (cell Tor19, Fig. 2*B*), had a sign-reversed pattern of correlations across the brain compared to the first neuron, owing to nearly anticorrelated spiking fluctuations. Inverse polarity maps displayed by neighboring neurons were a common feature across the recordings. The full brain maps for the two example neurons are shown in *SI Appendix*, Fig. S2 *A* and *B*. As cell Tor10 was judged to be stable across several weeks, *SI Appendix*, Fig. S2*C* shows the fMRI maps obtained over multiple sessions. To aid visualization, we computed surface maps of these correlations, including focused analysis of a strip of cortex in the superior temporal sulcus (STS; Fig. 2*C*). The correlation maps for cell Tor10 computed across all sessions are shown in the surface map in Fig. 2*D*.

We next asked to what extent neurons within a local population, in a sense occupying the same voxel, share their fMRI coupling with networks across the brain. This question was motivated, in part, by recent findings that during periods of natural visual behavior, neighboring face patch neurons differ markedly in their brain-wide fMRI coupling (24, 25). However, in contrast to naturalistic viewing conditions, we found that the spatial pattern of resting-state coupling was highly shared among neurons in a local population, aside from the polarity inversion exemplified in Fig. 2 *A* and *B* (see *SI Appendix*, Fig. S3). Comparing the cortical surface maps for the 79 unique AF neurons and 78 unique AM neurons revealed a high degree of spatial overlap in fMRI correlations (see all individual single unit fMRI maps in *SI Appendix*, Figs. S4 and S5). We summarized the consistency of these patterns across the population of neurons using two methods.

**Fig. 3.**
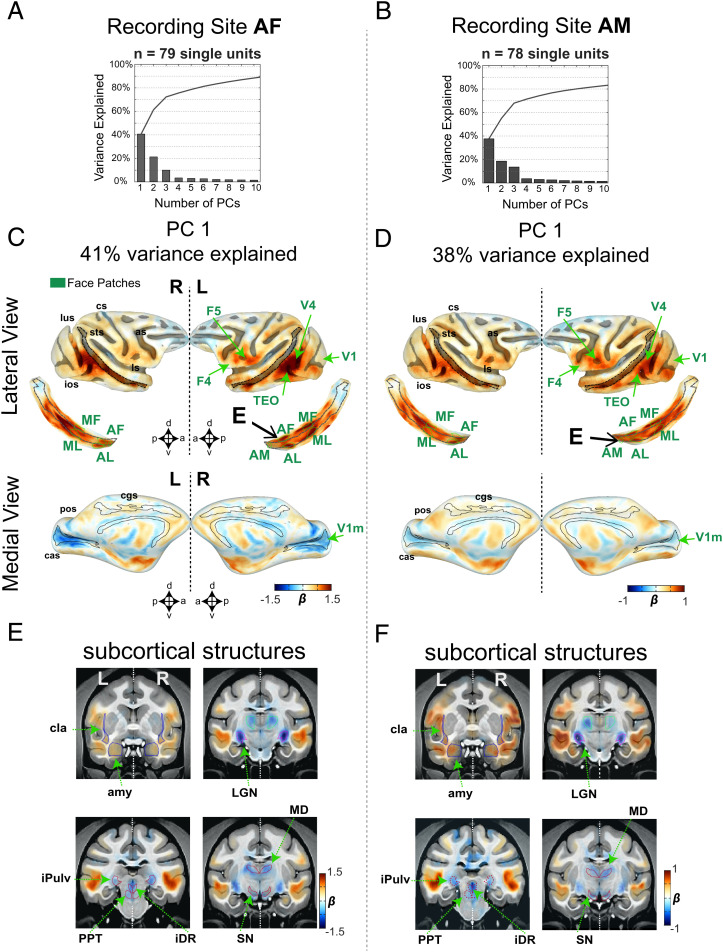
Population single unit fMRI response. Results from PCA across all neurons recorded in AF (*n* = 79 single units) and AM (*n* = 78 single units). Explained variance as a function of the number of principal components (PCs) for recordings in (*A*) AF and (*B*) AM. Functional activation maps corresponding to (*C*) recordings in AF and (*D*) recordings in AM are shown in the lateral (*Top*; along with the corresponding unfolded STS) and medial (*Bottom*) views of cortical surfaces. Cortical regions with strong association to the spiking activity are indicated by the green arrows. “E” indicates the location of the recording electrode. Functional activity in subcortical regions corresponding to (*E*) the first PC in AF and to (*F*) the first PC in AM. As in *C* and *D*, green arrows indicate subcortical regions with a strong correspondence to the spiking activity (see also *SI Appendix*, Figs. S4 and S5 for all individual single unit fMRI maps in each monkey and *SI Appendix*, Fig. S8 for ROI names). amy, amygdala; cla, claustrum; iPulv, inferior pulvinar; MD, mediodorsal thalamus. See *SI Appendix*, Figs. S6 and S7 for results corresponding to the second and third PCs.

First, we performed principal component analysis (PCA) on the single unit fMRI maps to assess the shared brain-wide functional connectivity stemming from different neurons. Functional maps were first collapsed into linear vectors composed of 90,112 voxels. The vectors from all neurons served as input to a standard PCA algorithm, with data from face patch AF and AM analyzed separately (Fig. 3). This analysis revealed that the first principal component carried a high proportion of the explained map variance across each neural population. The PCA maps corresponding to the first eigenvector are shown for neurons in AF (41% variance explained, Fig. 3 *C* and *E*) and in AM (38% variance explained, Fig. 3 *D* and *F*). The maps from the two face patch populations were highly similar, despite the spatial separation of the recorded neurons and the known functional distinctions between the face patches (21, 31). For neurons from both patches, the strongest coupling to the fMRI signal was found bilaterally in other temporal cortex face patches, in cortical areas V4, TEO, and somewhat less in ventral-premotor areas (F4 and F5), STS regions outside the face patches, and areas 45 A/B. Note that positive scores shown throughout the cortex in the PC1 maps capture neurons having maps of both positive and negative polarity in these regions, since the generation of the eigenvector is blind to the absolute sign. Beyond the cortex, weaker fMRI correlations were observed elsewhere in the telencephalon, including the amygdala, claustrum, and hippocampus (Fig. 3 *E* and *F*).

Other brain structures showed an inverse relationship to the cortical regions featured above. Most prominently, the LGN stood out in having highest negative coefficient in both face patches (Fig. 3 *E* and *F*; see also *SI Appendix*, Fig. S2*A*). This finding highlights an unexpected coupling between face patch neurons and the LGN during rest. A negative coefficient was also found in several brainstem neuromodulatory centers such as the substantia nigra (SN), pedunculopontine tegmental area (PPT), and dorsal raphe (iDR), respectively associated with ascending dopaminergic, cholinergic, and serotonergic projections. This consistent pattern of inverse correlations among subcortical structures, as revealed in the first eigenvector, was not observed previously with conventional seed-based rs-fMRI (3, 8). The correlational structures revealed by the second and third eigenvectors were less pronounced (*SI Appendix*, Figs. S6 and S7). The second eigenvector showed a shared correlation affecting a broader portion of the medial occipital cortex together with the STS, a finding that was more evident in the maps extracted from AF than AM.

**Fig. 4.**
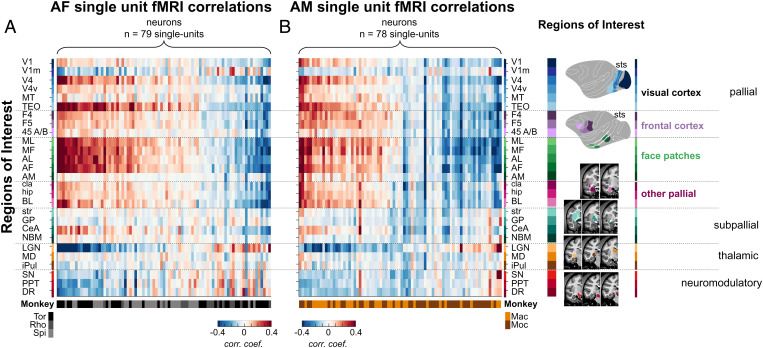
Similarity of single unit fMRI activation maps for AF and AM. fMRI correlation profiles across 27 functional regions of interest for each recorded single unit in (*A*) AF and in (*B*) AM. Each column (*n* = 157) represents a vector of 27 correlation coefficients from one neuron. All neurons are grouped based on their correlation profile similarity and monkeys from which they were collected. ROIs are listed and color coded according to their anatomical locations within the subdivisions of the standard developmental anatomical brain (*Right*), namely pallium (including visual areas, face patches, frontal areas; BL, basolateral amygdala; hip, hippocampus), subpallium (CeA, central amygdala; GP, globus pallidum; NBM, nucleus basalis of Meynert; str, striatum), thalamus (iPul, inferior pulvinar), and brainstem neuromodulatory centers (DR, dorsal raphe). See *SI Appendix*, Fig. S3 for the proportion of cells with positive and negative correlation maps and *SI Appendix*, Fig. S8 for the ROI names and their spatial extent.

For the second assessment of fMRI coupling across the population, we applied a region of interest (ROI) analysis across the brain. The ROIs were restricted to those areas showing consistent positive or negative values in the PCA results. Fig. 4 shows the raw correlation coefficients of all neurons in the study for each ROI, shown separately for AF and AM (Fig. 4 *A* and *B*, respectively; see *SI Appendix*, Fig. S8 for ROI details). For each face patch, the neurons are ordered based on their correlation strength. At the bottom of each column, the coloration of the horizontal bar indicates the monkey subject from which each neuron was measured. This ROI-based visualization highlights key features of the data. First, the correlation patterns and the set of areas involved were broadly similar across the two recorded patches in the five monkeys. Second, while neurons differed in the polarity of their correlations, the relative polarity between the ROIs was generally consistent. For example, most neurons had opposite sign correlations between the cortical face patches and subcortical neuromodulatory centers. Finally, the unexpectedly strong correlation of face patch neurons to the LGN, opposite in sign to the correlation with most cortical areas, was a common feature in both face patches and across all monkeys. Together, these findings demonstrate that face patch neurons show a high level of overlap in their spontaneous coupling to a restricted set of cortical and subcortical areas. In the next sections we examine these findings in greater detail.

### Hemispheric Asymmetry in Single Unit Connectivity.

**Fig. 5.**
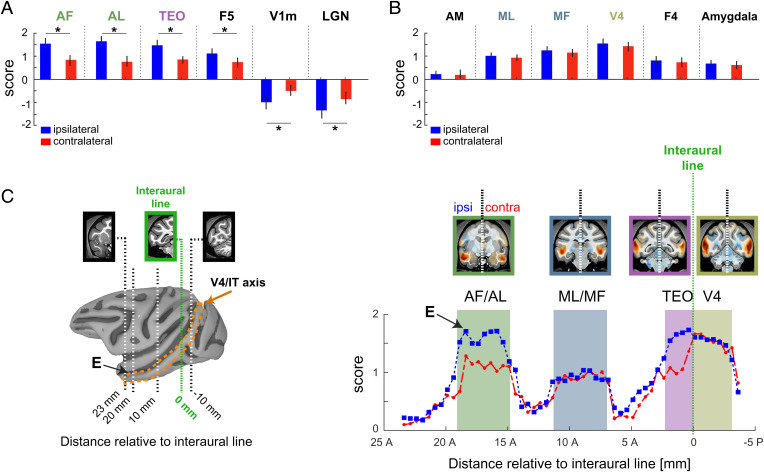
Interhemispheric differences in fMRI coupling to spiking activity from AF. Difference in functional activity between the recording hemisphere, or ipsilateral, and the contralateral hemisphere. (*A*) Comparison between the ipsilateral (shown in blue) and the contralateral (shown in red) hemispheres from regions showing a significant difference in the strength of fMRI association to the spiking activity. (*B*) Regions with no evident difference across the two hemispheres. (*C*) Distribution of the fMRI association strength to the spiking activity along the V4/IT axis (*Right Panel*). Shaded colored areas denote the relative spatial extent of visual cortical areas along V4/IT axis. The spatial extent of the V4/IT axis is indicated by the orange dotted lines in the cortical surface (*Left Panel*). *X*-axis in either panel depicts the coronal slice number relative to the interaural plane (dotted green line). “E” indicates the relative location of the recording electrode. See *SI Appendix*, Fig. S9 for results in AM.

We next investigated the relative strength of the fMRI coupling ipsilateral and contralateral to each recorded neuron, given that the recorded neurons each stemmed from either the right or left hemisphere and that the corpus callosum provides an incomplete set of interconnections between the two hemispheres (32). This analysis revealed that some fMRI regions showed stronger correlations to neurons on the side of the recordings, whereas others were equal in the two hemispheres. Specifically, the ROIs depicted in Fig. 5*A* showed a significantly stronger correlation on the side ipsilateral to neurons in the AF recording site than contralateral (*P* < 0.05 paired *t* test). This hemispheric difference was strongest in fMRI signals from the anterior face patches (AF and anterior lateral), where the strength in the ipsilateral side was nearly double that of the contralateral side. An ipsilateral bias was also evident in the V1m and LGN, structures that exhibited an inverse coupling. For other ROIs showing strong fMRI coupling, the difference between the two hemispheres was not significant (Fig. 5*B*, *P* > 0.05, paired *t* test). Comparing the ipsilateral and contralateral fMRI coupling strength along the anterior–posterior axis of the STS revealed a continuously varying level of hemispheric bias (Fig. 5*C* and see *Materials and Methods*). Similar asymmetries were observed in the maps derived from neurons in the AM face patch (*SI Appendix*, Fig. S9).

### Comparison of Single Unit, LFP, and fMRI Seed Maps.

**Fig. 6.**
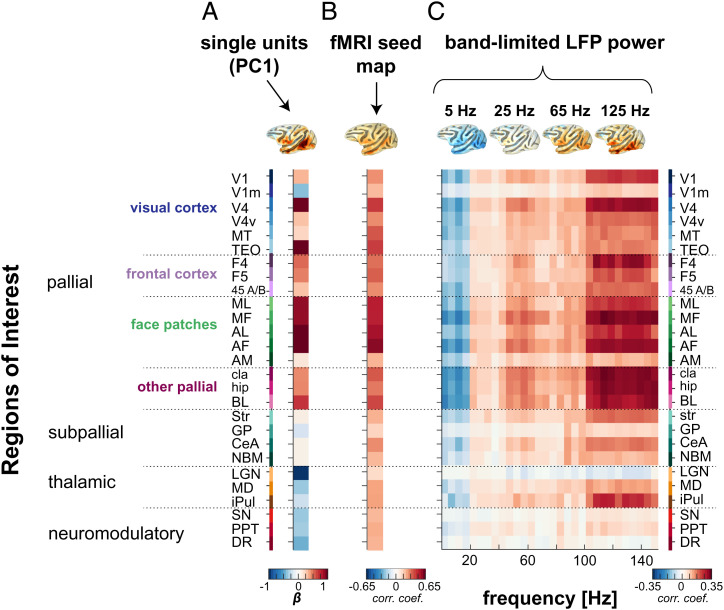
Comparison between spiking, fMRI, and LFP activity from AF face patch. Profile of fMRI activations from (*A*) the population spiking activity with (*B*) the averaged correlation profile associated to the fMRI seed in AF (seed across *n* = 20 scans) and with (*C*) the correlation profiles across different LFP frequency bands, where each column represents a vector of 27 correlations across LFP frequencies spaced by 5 Hz each (*n* = 30 columns representing 30 LFP frequencies). The correlation profiles are accompanied by their corresponding functional map plotted on a brain surface (*Top Panel*). The listed ROIs are ordered similarly as in Fig. 4 (see *SI Appendix*, Fig. S8 for the ROI names and their spatial extent; also see *SI Appendix*, Fig. S10 for functional maps corresponding to each signal modality and *SI Appendix*, Fig. S11 for results in AM).

How do single unit fMRI maps compare to those computed from other signals measured simultaneously from the same voxel, such as the LFP or hemodynamic time course? To address this question, we repeated the seed-based correlation analysis for AF, comparing the ROI profiles from the first principal component of the single unit activity (Fig. 6*A*) with those derived from the local fMRI signal (Fig. 6*B*) and from the power fluctuations in different frequency bands of the LFP (Fig. 6*C* and *SI Appendix*, Fig. S10 and see *SI Appendix*, Fig. S11 for corresponding results from AM). The maps derived from the different signal types overlapped with the single unit maps in their correlation patterns, particularly in the cortical correlations derived from the fMRI seed and high-gamma (>100 Hz) LFP seed. At the same time, there were important differences. For example, the fMRI seed produced exclusively positive correlations, with the face patch network showing the highest correlations, consistent with previous findings (8). The strong, inverted coupling with the LGN, neuromodulatory centers, and V1m were absent entirely. There was also little evidence of inverse coupling within LFP power fluctuations, beyond the global reversal in polarity for frequency bands below and above 20 Hz (33). However, one potentially important exception to this rule was the LGN, where fMRI fluctuations exhibited a weak, negative correlation with gamma-range LFP signals that was opposite in sign to the fMRI correlations within the cortex. Nonetheless, the overall pattern of fMRI correlation derived from single units did not closely match that derived from the local hemodynamic or LFP-based signals, particularly in subcortical structures. This lack of correspondence underscores the inherent differences in the information carried by local signals measured from the same voxel (12).

## Discussion

The intrinsic correlational structure of spontaneous fMRI activity is routinely used to study large-scale networks in the human brain (3, 4, 34). Electrophysiological and imaging experiments in animals demonstrate that such correlations are expressed at multiple spatial and temporal scales (10, 14, 35–37) and vary with brain state and neuromodulation (2, 34, 38, 39). We used simultaneous fMRI and single unit recording in the macaque to assess the intrinsic brain-wide coupling exhibited by neural populations in two functionally defined face patches located at different positions in the temporal cortex. We used the monocrystalline iron-oxide nanoparticle (MION) contrast agent principally because it is known to increase the functional contrast-to-noise ratio and thus the estimation of the spontaneous correlation with individual neurons. We found that neurons in both areas were coupled with fMRI fluctuations in a distinct set of brain regions. These included circumscribed areas of the temporal lobe, including STS face patches and parts of TEO, which may overlap with the PL face patch; early visual cortex, most notably area V4 and V1m; regions in the ventral premotor cortex; visual thalamus, including LGN and iPul; and brainstem neuromodulatory centers. The spontaneous correlations were highly similar among neurons in AF and AM, although there were some differences, for example in the strength of their correlations with V1 and with the amygdala. While these differences were subtle compared to the common features, they probably reflect unique connections and functional specializations of individual face patches.

A subset of the observed fMRI correlations follows logically from known connections of the face patch system. For example, the single unit coupling with other face patches, the amygdala, claustrum, and hippocampus, is consistent with measures of connectivity based on tracer injections (22), microstimulation (23), and resting state fMRI connectivity (8). Moreover, the asymmetry of some fMRI correlations, which has also been observed in seed-based fMRI (40), may be understood in terms of known interhemispheric connections (32). For example, the right and left middle lateral patches showing symmetrical coupling have direct hemispheric connections, whereas the anterior face patches showing asymmetric coupling do not (22).

Other patterns of fMRI correlations are more difficult to reconcile with straightforward anatomical explanations, including some in the cerebral cortex. For example, the prominent correlation with V4 and the STS correlations between the face patches are not expected based on previous anatomical or microstimulation studies, which indicate that face patches are primarily interconnected with one another (22, 23). Similarly, the ventral premotor cortex does not have a direct anatomical pathway. The functional connections with the ventral premotor cortex are intriguing, as they may pertain to understanding facial actions (41). However, the most likely anatomical route of communication is through a relay in the posterior parietal cortex (42), although no fMRI correlations were observed in that region. Thus, for some cortical areas the observed correlations may emerge indirectly, for example through common input from other visual areas or neuromodulatory centers.

The inverse fMRI correlations observed in the SN, iDR, and PPT may derive from known projections of brainstem neuromodulatory centers to the temporal cortex, including TEO and TE (43). Such projections could directly or indirectly influence the firing of neurons within face patches, for example by generating distinct spatiotemporal modes of cortical activity (2, 44). Most surprising, however, was the strong, inverse coupling of face patch neurons with the entire LGN of both hemispheres. While anatomical projections linking the temporal cortex and LGN have been reported, current evidence suggests that they are extremely sparse, particularly in the adult (43, 45), although this is an active topic of investigation (46, 47). A more likely explanation is that the LGN correlations stem from changes in brain states conferred to the thalamus by neuromodulation, such as cholinergic modulation stemming from the PPT (48, 49). During normal visual behavior, afferents from this brainstem nucleus are thought to mediate state-dependent changes in the processing of visual information in the LGN (50), thus controlling the magnitude of visual input to the cortex (51, 52). A similar explanation may relate to the iDR, which also projects to the LGN (44, 53) and which in our findings also showed inverse correlations. Neuromodulatory signals, perhaps through the actions on the LGN, may also explain the concurrent negative coupling observed in the V1m, although it does not explain why only the medial portion is affected. Further studies are needed to understand the strong coupling in the LGN, its relationship to specific recording sites, and whether this functional connection might have any bearing on normal visual function.

A striking feature of the data was the mixing of neurons having correlation with the same brain areas but with opposite polarity. Approximately one third of the neurons we recorded exhibited this inverse correlation pattern, which could be traced to inverted time courses of spiking fluctuations. Previous work in the mouse has found anticorrelated networks among neighboring neurons that fluctuated over similar time scales (54, 55), with two anticorrelated networks at least partially segregated into different layers. While our microelectrode bundle spans less than 1 mm, we cannot rule out the possibility that the populations of anticorrelated neurons are at least partially segregated across layers. However, it is notable that we found similar ratios of neurons with positive- and negative-correlated face patch correlations in each of the monkeys tested (*SI Appendix*, Fig. S3).

In rs-fMRI, the influence of arousal systems is well acknowledged, for example with the basal forebrain thought to contribute prominently to the shared fMRI signal fluctuations, commonly known as the global signal (38, 56). These influences have typically been considered through their widespread effects on hemodynamic signals, whose relevance to functional connectivity is debated (14). The present study demonstrates that individual neurons in the high-level visual cortex carry signals ostensibly related to arousal. These signals were expressed through their selective correlations with neuromodulatory centers and structures such as the LGN and V1m. The same neurons did not show spiking correlation broadly across the cortex or thalamus, raising new questions. First, how does the neuromodulatory contribution to a given cortical neuron have such restricted pattern of areal covariation that does not involve other regions that are also regulated through arousal fluctuations? Second, why would strong, inverted correlations with neuromodulatory centers and the LGN be revealed through neural–fMRI correlations but not through fMRI–fMRI correlations? One potential answer to these questions is that the local integration of excitatory, inhibitory, and modulatory inputs to a given neuron during rest confer a regional identity that decouples its spiking activity from that of neurons in other brain areas, even as they are all subject to coordinated brain state changes. Since the two face patches in the present study occupied the same broader network, addressing this question may require repeating these experiments for neurons occupying different brain networks. One might ask, for example, whether the spontaneous activity of neurons in the frontal cortex is also selectively coupled with the LGN or, perhaps, is more closely related to another thalamic nucleus such as the mediodorsal nucleus.

It is interesting to contrast the present findings with recent work in which the time course of face patch neurons was compared to whole-brain fMRI activity during periods of naturalistic visual experience (24, 25). Those previous studies did not measure the simultaneous covariation of spontaneous signals but rather the driven covariation of externally visual inputs. Nonetheless, the difference in fMRI network correlations to the present study is striking, particularly given that the recordings were carried out from the same face patches. Most notably, in contrast to the present study, the neurons in the previous study showed strong positive and negative coupling with a much broader range of brain regions. Individual cells in a local population were highly diverse in their network affiliations. Importantly, many of the structures showing covariation with face patch neurons during free viewing did not show any significant spontaneous coupling at rest in the present study. Conversely, the inverted coupling of the LGN and neuromodulatory centers revealed here were not observed in the previous study.

While the current study did not aim to address neurovascular coupling directly, it is important to note that the widely shared pattern of coupling exhibited across the single neuron’s population was not directly reflected in the power of any of the LFP frequency bands we analyzed. In contrast to spiking correlations, the correlations of each LFP band across areas were of the same sign, with the absolute polarity varying as a function of frequency band (33). One exception to this was some weakly inverted signals, most notably in the correlations of the LGN with the LFP. In general, the correlations of the high-gamma LFP showed closest agreement with those of the single units, partially supporting their utility as a proxy to spiking activity (12, 57).

Hence, fMRI mapping of individual neuron activity has the capacity to tap into multiple different aspects of brain function. Importantly, it allows researchers to relate local activity, whether spontaneous or task based, to patterns of action across the brain. The present study assessed the homogeneity of single unit fMRI maps, finding ∼40% of the variance shared across the population. Whether this is a high or low level of overlap may depend on one’s perspective. In comparison to analogous mapping during natural modes of vision, the maps are highly homogeneous and restricted in their scope of correlations. Future work will continue the effort to understand the rich information that can arise through the multimodal analysis of brain-wide activity covariation, including unique information afforded by different neural activity measures, time scales, and task conditions.

## Materials and Methods

### Subjects and Ethical Statement.

We used five male rhesus monkeys (*Macaca mulatta*) weighing 7–9 kg. All animals were surgically implanted with a custom-designed MR-compatible head post and MR-compatible chronic microwire electrode bundles targeting face patches (26) (Fig. 1*A* and *SI Appendix*, Fig. S1; AM face patches for two animals, AF face patches for three animals). All surgeries were carried out under general anesthesia with isoflurane and were approved by the Animal Care and Use Committee of the US National Institute of Mental Health/National Institutes of Health. After surgery, animals were given analgesics and prophylactic antibiotics. During experiments, the animals were on water control and received their daily fluid intake during their testing. Each subject’s weight and hydration level were monitored closely and maintained throughout the experimental testing phases. All the experimental procedures were in full compliance with the Guidelines for the Care and Use of Laboratory Animals by US National Institutes of Health.

### MRI Scanning.

Structural and functional MRI experiments were acquired on a vertical 4.7-T Bruker BioSpec scanner with 60-cm-diameter bore magnet (Bruker BioSpin GmbH, Germany). The Bruker S380 gradient coil had a maximal slew rate of 340 mT/m/s and a maximal strength of 56 mT/m. Animals sat upright in a specially designed chair. The chair base was equipped with a module housing an electromagnetic interference filter (see *Neuronal Recordings* section). Whole-brain images were collected with either an eight-channel receive radiofrequency coil system (RAPID MR International, Columbus, OH) or a custom-made four channel coil. A total of 67 fMRI sessions (39 sessions with an electrode in AF and 28 sessions with electrode in AM), combined with electrophysiology, were collected, and each consisted of 600 volumes. Each fMRI experiment session was acquired with a single-shot gradient echo planar imaging (EPI), slice thickness of 1.5 mm, an in-plane resolution of 1.5 × 1.5 mm^2^, and a matrix of 44 × 64 × 32. Echo time and TR were 12 ms and 2,000 ms, respectively. All functional volumes in our experiments were acquired at the beginning of each TR. This was essential for our electrophysiological experiments as the gap between volume acquisition provided us with 1.2 s time window of MRI artifact-free signal.

### MION.

Prior to the start of EPI data acquisition, we intravenously administered MION. MION is a T2* contrast agent that isolates functionally related changes in cerebral blood volume, primarily in the arterioles. MION was used because of its high contrast-to-noise ratio and is common practice in monkey fMRI (58–60). We obtained MION from the Imaging Probe Development Center, National Heart Lung and Blood Institute, Bethesda, Maryland. We determined the dosage of MION by monitoring the drop in intensity in the brain following injection. Previous work has shown that a drop of roughly 50% is optimal for good functional imaging, and this corresponded to roughly 8–10 mg/kg MION, depending on the monkey and batch of MION. Note that, in contrast to the BOLD signal, activity-based increases using fMRI MION signals (also known as regional cerebral blood volume) are visible as decreases in signal intensity. For the sake of clarity, we therefore inverted the sign of modulation throughout the article.

### fMRI Data Processing.

fMRI data were analyzed using the AFNI/SUMA software package (61) and custom-written MATLAB code (MathWorks, Natick, MA). Prior to the analysis, all raw images were first converted into AFNI data file format (BRIK and HEAD files). Slice timing was corrected to be alternating in the z+ direction using the AFNI function 3dTshift with an option of using the quintic (fifth-order) Lagrange polynomial interpolation. Motion correction algorithms were applied to each EPI time course with the AFNI function 3dvolreg. Magnetic field distortions were corrected with the PLACE algorithm (62). Each 30-min scan was converted into the percentage signal change by subtracting the mean and then dividing by the mean. Subsequently, each session was then registered to a template session, allowing the combination of data across multiple testing days.

### Neuronal Recordings.

Each experimental day, the setup for the simultaneous recordings involved restraining the head of the monkey and plugging the amplifier cable into the implanted connector, a procedure requiring just a few minutes. The monkey chair was equipped with an MRI-compatible electromagnetic interference filter (high-performance D-sub filter, APITech’s Series 700 EMI) which helps to reduce the electromagnetic interference. The electrodes consisted of bundles of 32 NiCr or 64 NiCr microwires chronically implanted in the AM and AF face patches (26). The microwire electrodes were designed and initially constructed by Dr. Igor Bondar (Institute of Higher Nervous Activity and Neurophysiology, Moscow, Russia) and subsequently manufactured commercially (Microprobe for Life Science, Gaithersburg, MD). The ground and reference were connected to one another and to a designated reference wire within the microwire bundle, as well as to a gold-plated grounding terminal adjacent to the dura, using a ceramic screw placed ∼1 cm from the implant margin. The signals obtained inside the 4.7-T scanner were amplified and digitized at 24.4 kHz with a PZ5 NeuroDigitizer (TDT) and then sorted to an RS4 Data Streamer controlled by an RZ2 BioAmp Processor (TDT). The TDT system has a resolution of ∼25 kHz and 250 nV/bit with an input range of ±500 mV, which allowed us to capture the entire electrophysiological signal with no saturation induced by the MRI artifact. Only segments of electrophysiological activity recorded during the 1.2-s gap between volume acquisitions were considered for the analysis. However, it is worth mentioning that that even during these periods of MRI artifact-free signals, large residuals were still noticeable in our recordings. Therefore, we needed to account for the residuals by capturing the differences from the averaged waveform. For this purpose, we used PCA as described in previous studies combining fMRI and electrophysiology (63–65). The broadband electrophysiological responses were used to extract individual spikes in WaveClus software (66) after filtering between 300 and 5,000 Hz. LFPs were extracted from the broadband signal by fourth-order Butterworth bandpass filtering of the signals between 1 and 150 Hz with a sharp transition bandwidth (1 Hz). Forward and backward filtering were used to eliminate phase shifts (67).

### fMRI Correlations with Neural Activity and with Other Seed-Based Signals.

For each individual neuron isolated from AF and in AM, we computed the whole-brain functional maps across the entire 30 min of combined fMRI and electrophysiological recordings. The value of each voxel was the correlation coefficient between its fMRI time course and the single AF and AM neuron’s time course (Fig. 1*B*). To enable a direct comparison between the neuronal and fMRI measurements, we first down-sampled the time course of each single unit to match the fMRI temporal resolution. This was done by taking the spike count in bins of 1.2 s, which corresponds to the 1.2-s gap between volume acquisition described above. Subsequently, this new time series was convolved to a generic MION hemodynamic impulse response function (gamma probability density function) (68). Following these two preprocessing steps in the time courses of each single unit, we computed Spearman’s rank correlation coefficient between the neural time course and fMRI time courses of all the voxels in the entire brain (Fig. 1*B*). This procedure was applied to every single unit.

We exploited the stability of the microwire recordings to conduct longitudinal experiments over multiple days. The longitudinal identification of single neurons across days was based purely on waveform features and spike statistics (20). Details about this longitudinal detection of spikes across days have been described in detail elsewhere (26, 28, 29). According to our expectation, such stability was reflected in the characteristics of the spikes and in the stability of the fMRI maps across sessions as well (*SI Appendix*, Fig. S2*C*).

The functional maps that were derived from comparing fMRI time series with the LFP signals resulted from a similar procedure to the one we described earlier. The LFP signals in the current study were filtered into nonoverlapping frequency bins of 5 Hz, which were then rectified to create the band-limited power signal. This procedure was applied to all signals recorded from every microwire; however, subsequent LFP analysis was carried out only on microwires with a corresponding single unit. We refrained from using any convention to define LFP frequency boundaries as they reflect different neuronal processes (67) and have a distinct relationship to the fMRI signal (69).

We generated AF and AM seed voxel correlation maps, using one voxel close to the location of the electrode tip. This voxel, which serves as a seed, was then used to compute voxel-wise correlation between the time course of the seed voxel and all the voxels from the whole brain. The resulting maps remained the same whether we used other voxels close to the electrode tip.

All the correlation maps that resulted from this procedure were further aligned and projected onto the NMT macaque brain atlas (30, 70).

### Cross-Modal Coherence.

We quantified the cross-modal relationship between single units and fMRI by computing the coherence for 37 neurons in AF and 34 neurons in AM relative to the MION signals in areas showing the highest correlations. In total, six ROIs were evaluated, and the cross-modal coherence was computed across 852 voxels. Coherence estimates were computed as magnitude-squared coherence via Welch’s periodogram method, as shown in the formula depicted in *SI Appendix*, Fig. S12.

### Macaque Template Alignment and Atlas Parcellation.

Anatomical T1 sessions from each monkey were used to warp into the D99 atlas (71) and to the NMT (70). We used the @animal_warper program to generate all atlas files and create the output files necessary for the alignment of monkey anatomy to the NMT. The obtained D99 atlas ROI parcellations were used here as an initial template and subsequently modified based on the functional properties of each brain area and using images provided by CoCoMac macaque (cocomac.org). Subsequently the boundaries between regions were corroborated with the Paxinos macaque atlas (72). The generated ROIs were then interactively adapted into the NMT macaque atlas and were categorized into standard developmental subdivisions. For displaying the ROIs on the NMT brain surface, we first created a color mappable .niml file of the atlas volume with the command 3dVol2Surf. The activation results were displayed in SUMA along with ROI boundaries and contours.

## Supplementary Material

Supplementary File

## Data Availability

All study data are included in the article or *SI Appendix*.
