# Supplementary material for: Brain-wide functional connectivity of face patch neurons during rest
Source: Proc Natl Acad Sci U S A. 2022 Aug 31;119(36):e2206559119. doi: 10.1073/pnas.2206559119 (PMC9457296; doi:10.1073/pnas.2206559119)
Supplement: Supplementary File [file pnas.2206559119.sapp.pdf]

Supplementary Information for

Brain-wide functional connectivity of face patch neurons during rest

Daniel Zaldivar[1,*], Kenji W. Koyano[1], Frank Q. Ye[2], David C. Godlove[1], Soo Hyun Park[1], Brian E. Russ[1], Rebecca Bhik-Ghanie[1], David A. Leopold[1,2,*]

[1] Section on Cognitive Neurophysiology and Imaging, National Institute of Mental Health, Bethesda, MD 20892, USA.

[2] Neurophysiology Imaging Facility, National Institute of Mental Health, National Institute for Neurological Disorders and Stroke, National Eye Institute, National Institute of Health, Bethesda, MD 20892, USA

*Correspondence:

Daniel Zaldivar, E-mail: daniel.zaldivarperez@nih.gov or

David Leopold, E-mail: leopoldd@mail.nih.gov

This PDF file includes:

Supplemental Figures S1-S12

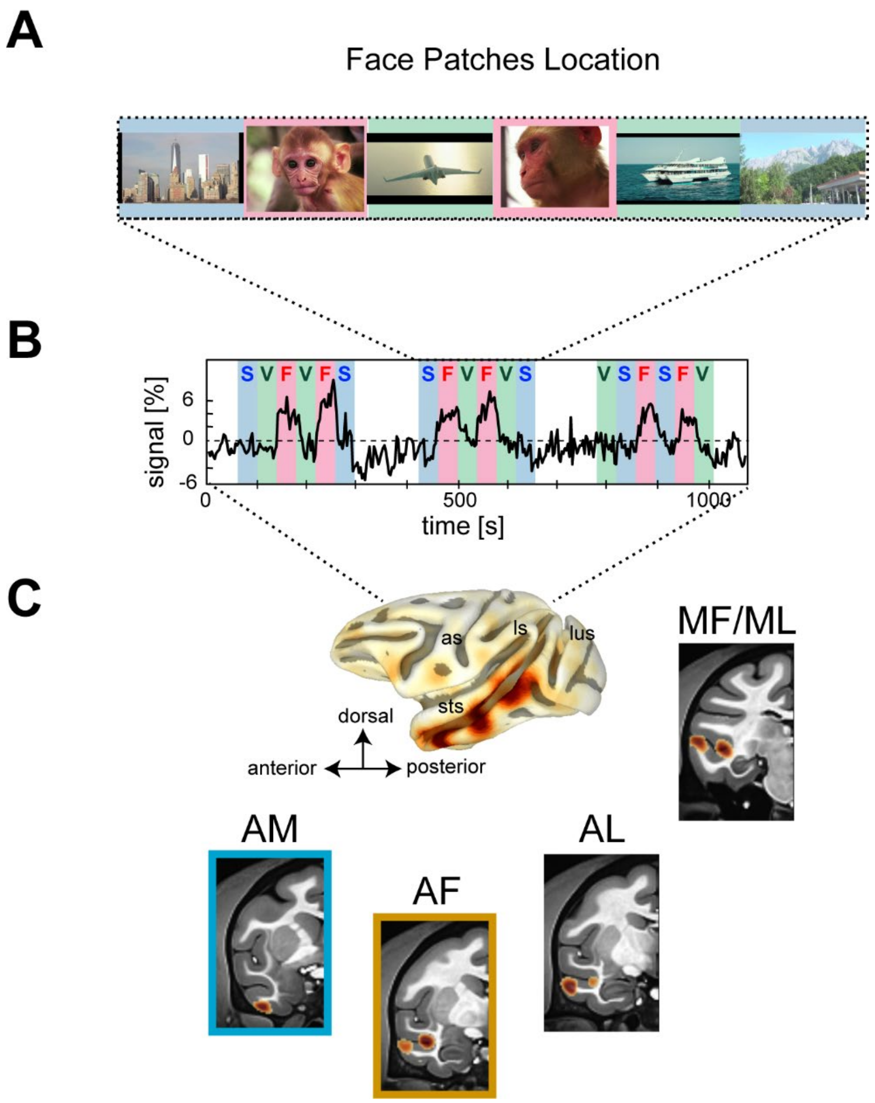

Fig. S1 fMRI targeting of face selective areas. **(A)** Schematic representation of the visual stimulation paradigm used for the fMRI-targeting of face patches. **(B)** fMRI response time series of a voxel located in the AF face patch showing selective response to the presentation of faces (shaded red area) in contrast to other objects (e.g., vehicles, shaded green area) or visual scenes (shaded blue area). **(C)** Cortical surface depicting the location of the face selective areas. Structural MRI and functional overlay showing activation in voxels corresponding to the face patch AM (anterior-medial), AF (anterior-fundus), AL (anterior-lateral), ML (medio-lateral) and MF (medio-fundus) face patch areas in monkey M4.

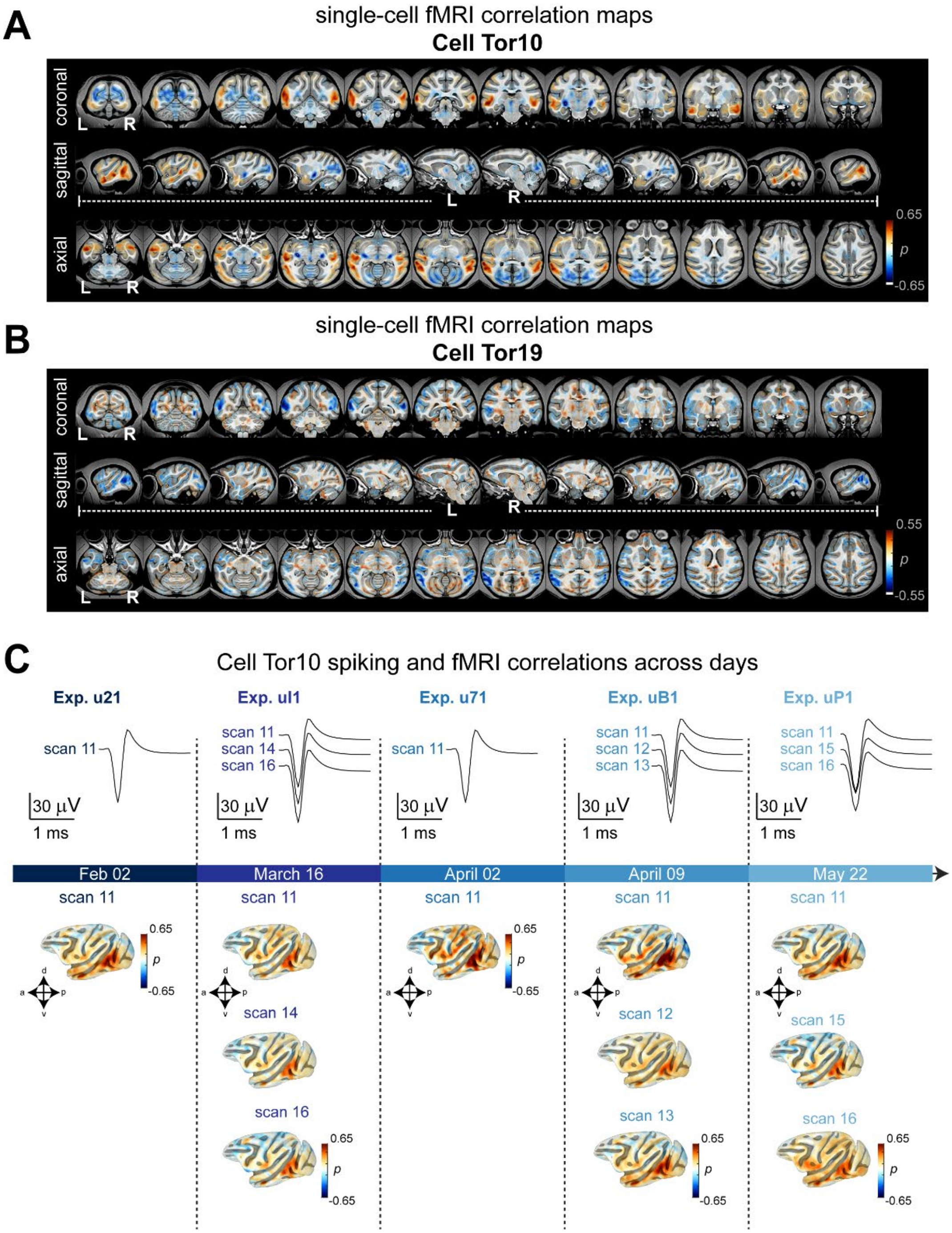

Fig. S2 Consistent single-unit fMRI maps across days. Single-unit fMRI maps from two example neurons recorded in the left AF. Activation maps for cell **(A)**, Tor10 and **(B)** cell Tor19, are displayed in the coronal (top), sagittal (middle) and axial (bottom) planes. **(C)** Consistency of single-unit fMRI activation maps from cell Tor10 collected across different scans and during multiple days. Each column depicts: the spike wave form from cell Tor10 recorded over the course of 4 months and during different scans (top); the cortical surface representation of the fMRI response from the recording hemisphere is depicted across days and scans (bottom). Dates of simultaneous fMRI and neurophysiology recordings are indicated in the blue rectangles.

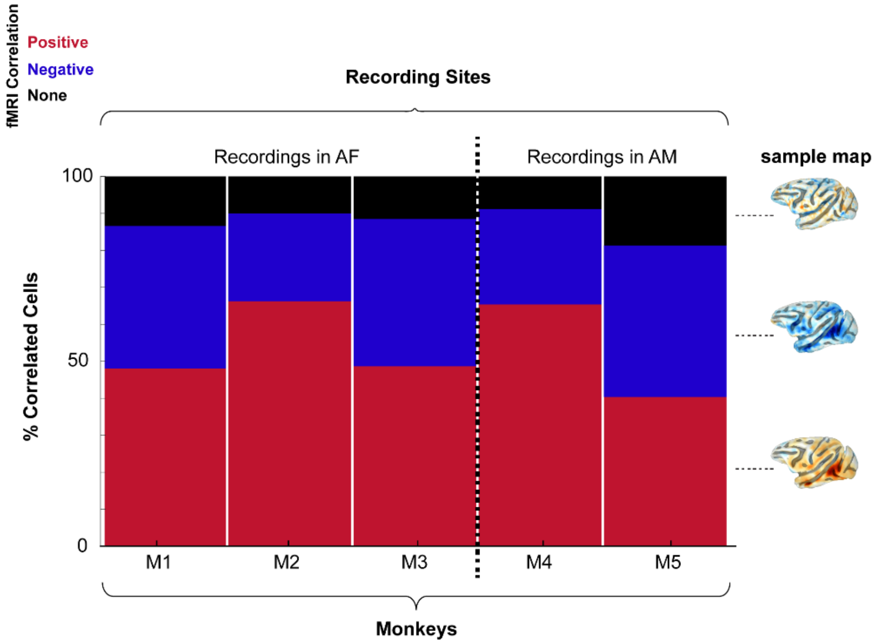

Fig. S3 Proportion of single-unit fMRI functional maps. Percentage of single-unit functional maps in each monkey with positive (red), negative (blue) and lack of correlational structure (black).

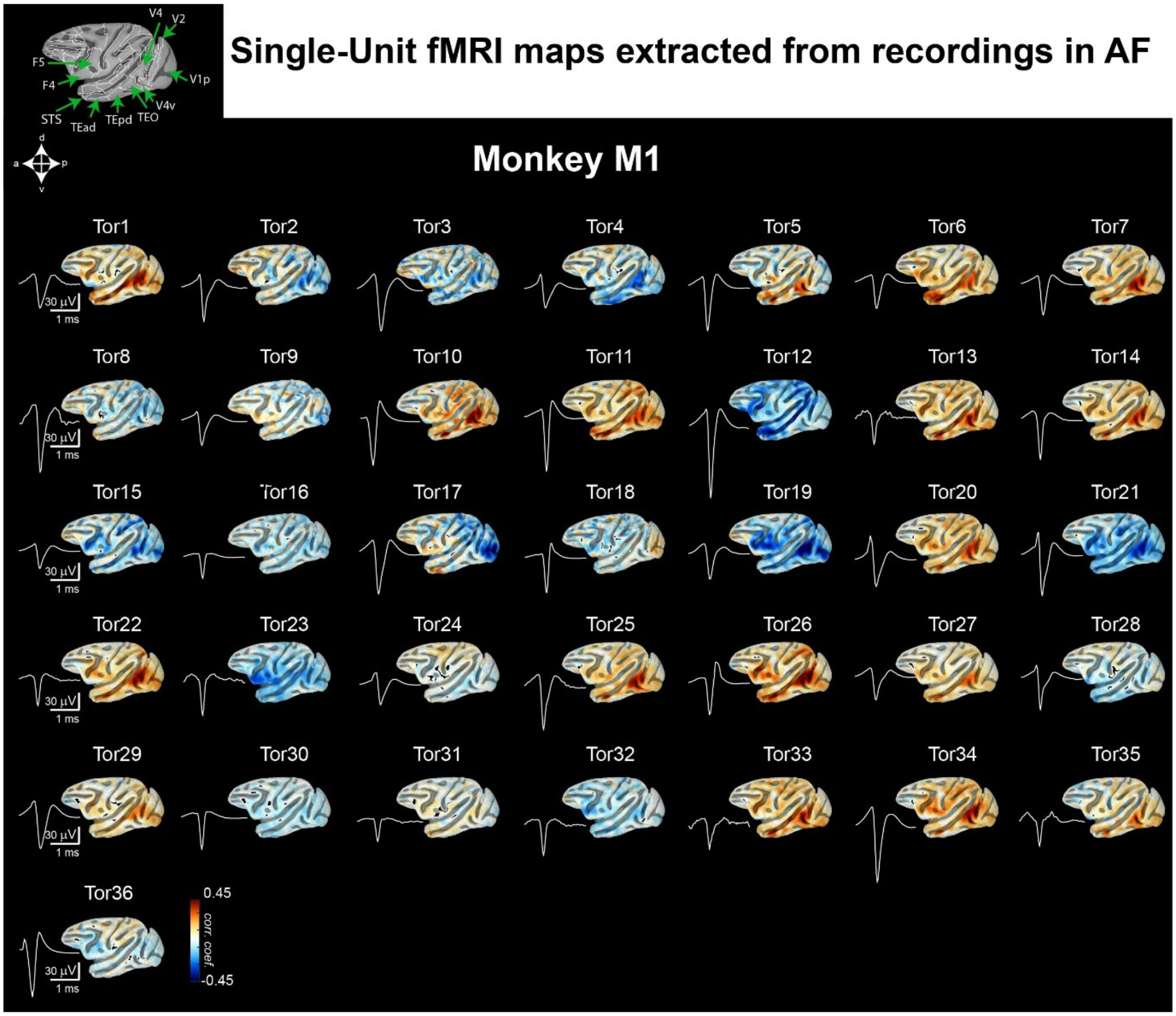

Fig. S4A Single-unit functional map from all single neurons recorded from monkey M1, accompanied by their respective spike wave form.

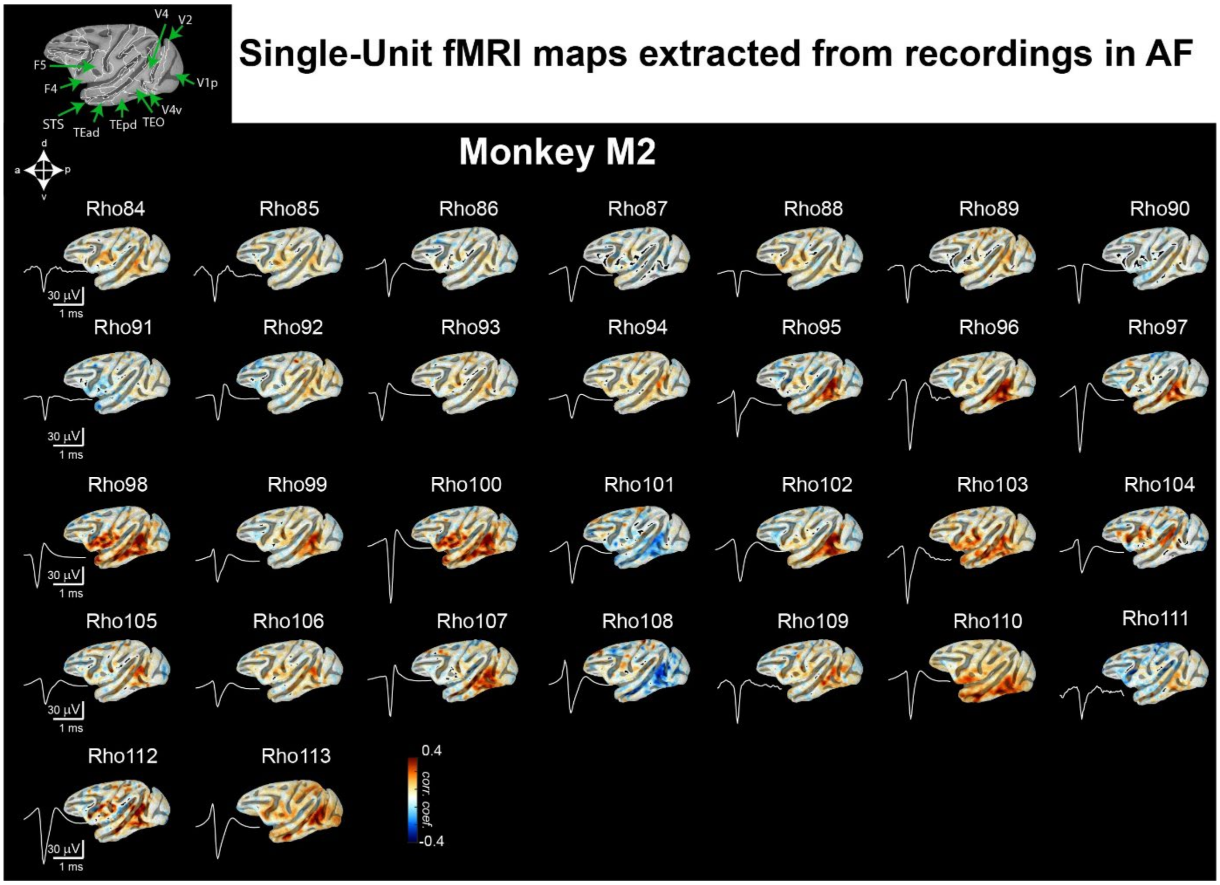

Fig. S4B. Single-unit functional maps from all single neurons from monkey M2 accompanied by their respective spike wave form.

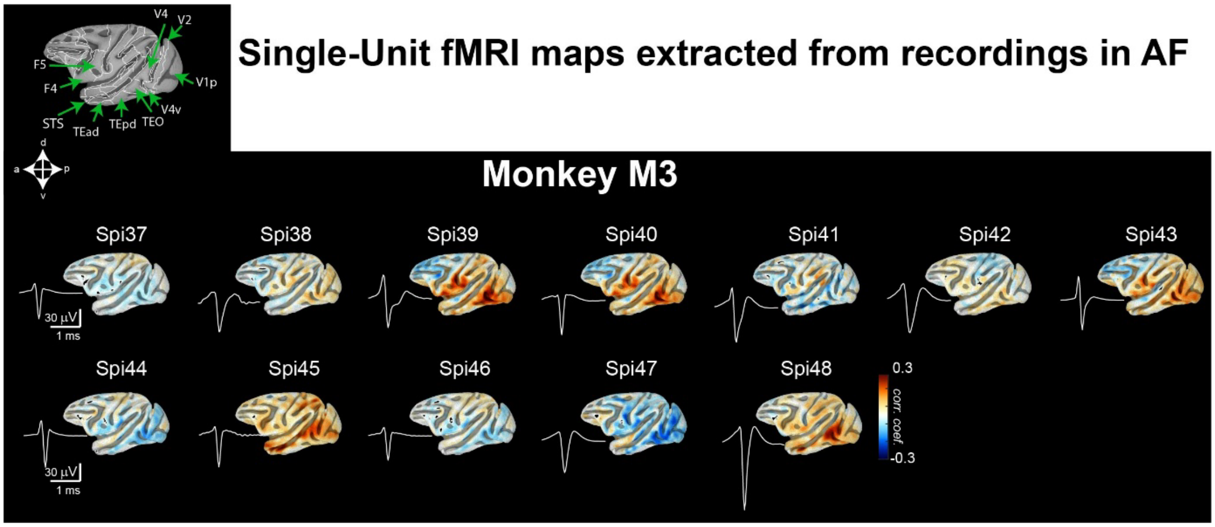

Fig. S4C Single-unit functional maps from all single neurons recorded from monkey M3, accompanied by their respective spike wave form.

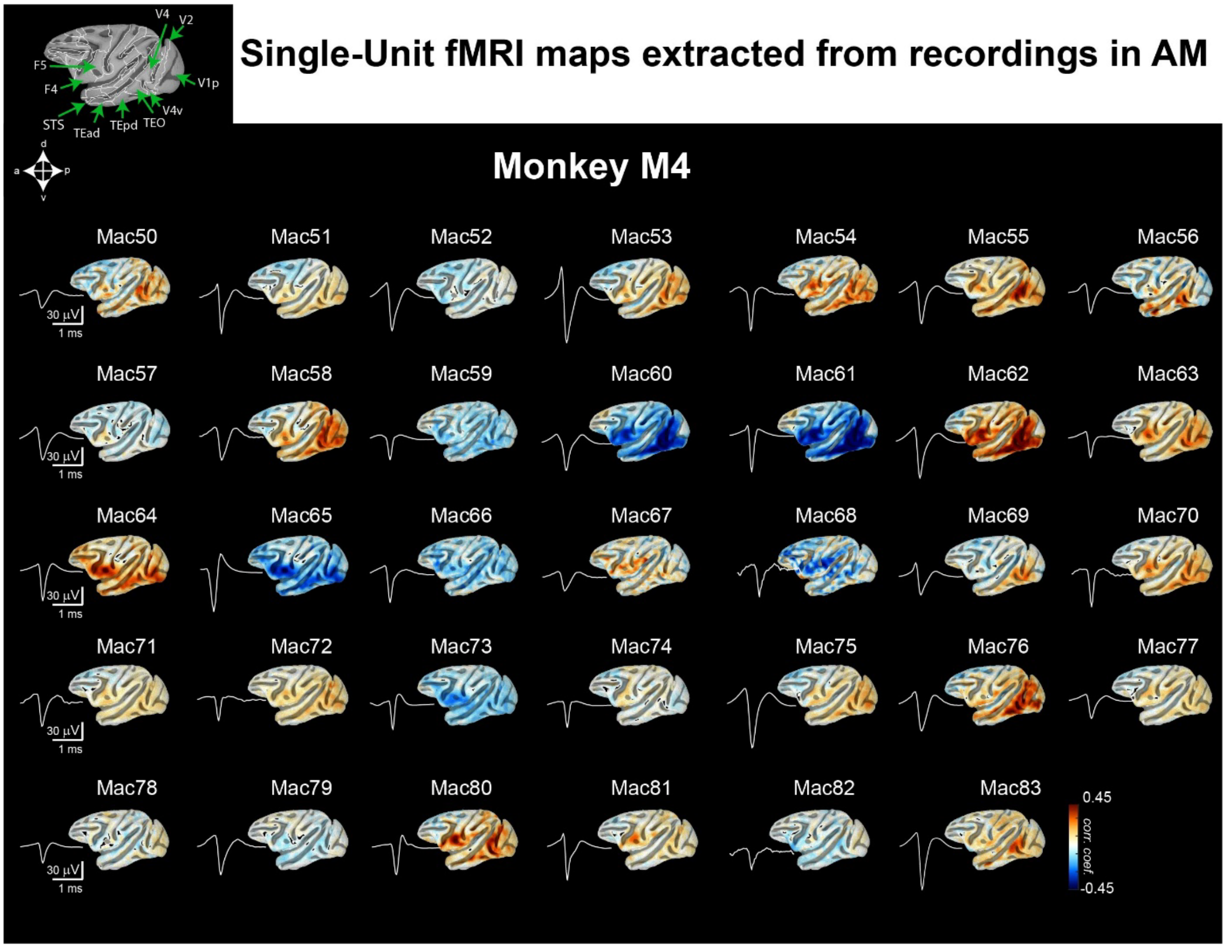

Fig. S5A Single-unit functional maps from all single neurons recorded from monkey M4, accompanied by their respective spike wave form.

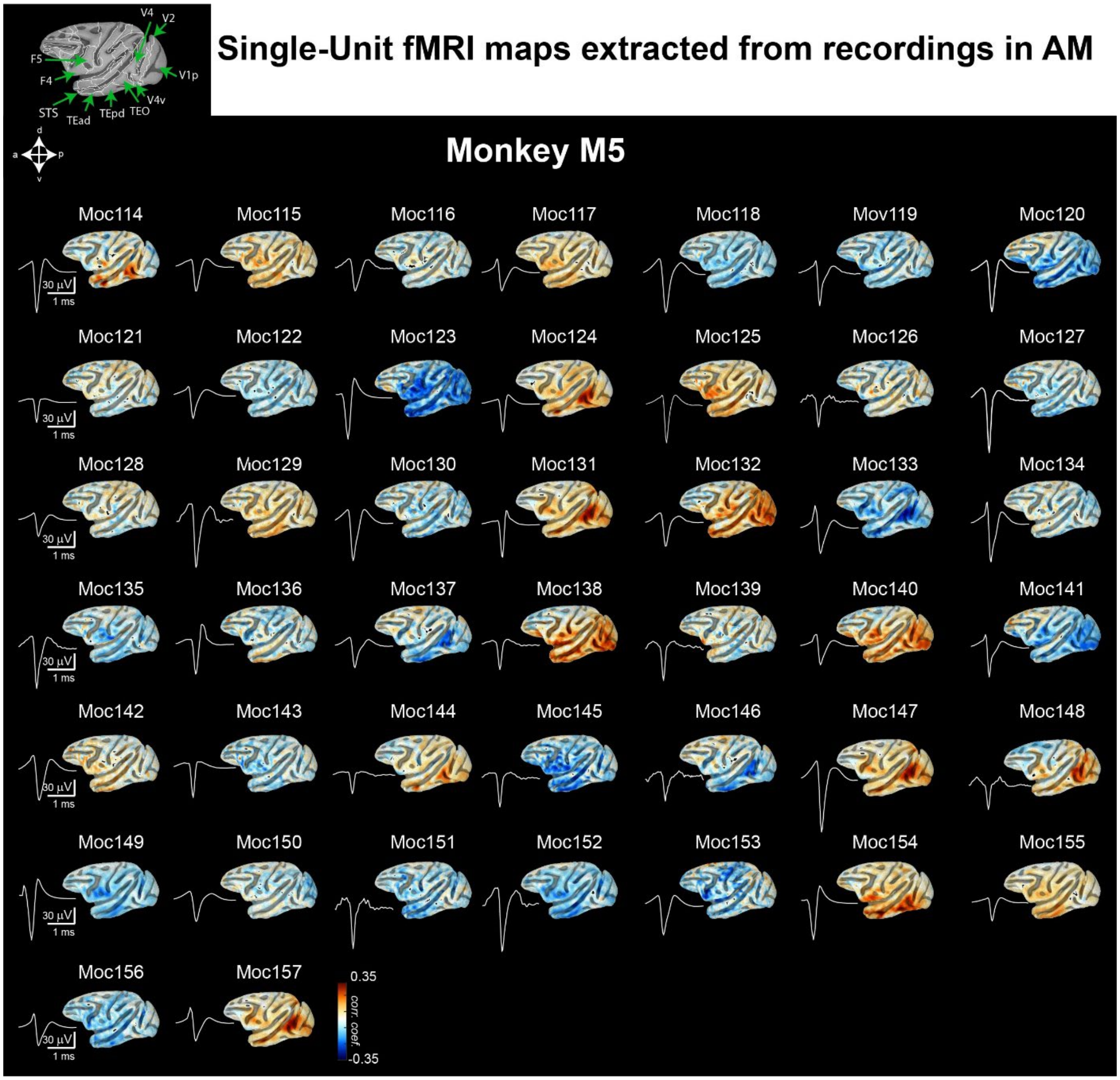

Fig. S5B Single-unit functional maps from all single neurons recorded from monkey M5, accompanied by their respective spike wave form.

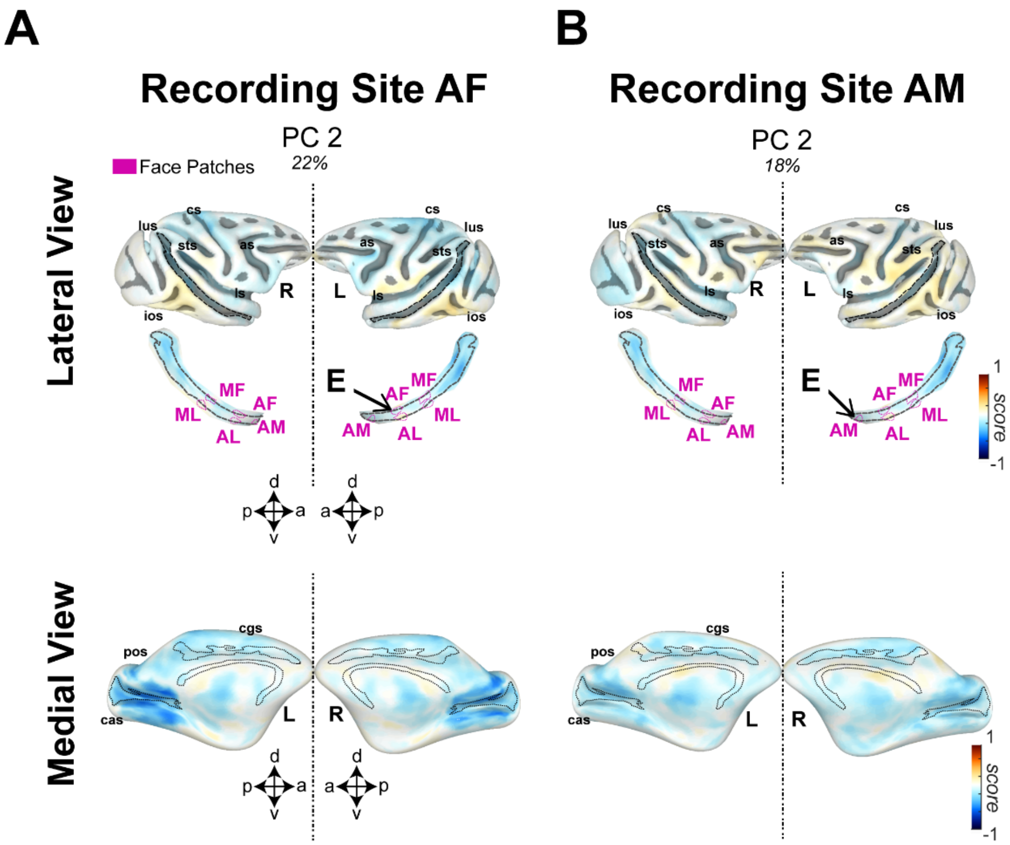

Fig. S6 Results from the second PC. Functional maps showing the results obtained from PCA analysis. Results are showing the second PC from recordings in **(A),** AF and in **(B),** AM. Lateral (top panel) and medial (bottom panel) views are shown for each corresponding recording site. “E” demarks the location of the electrode.

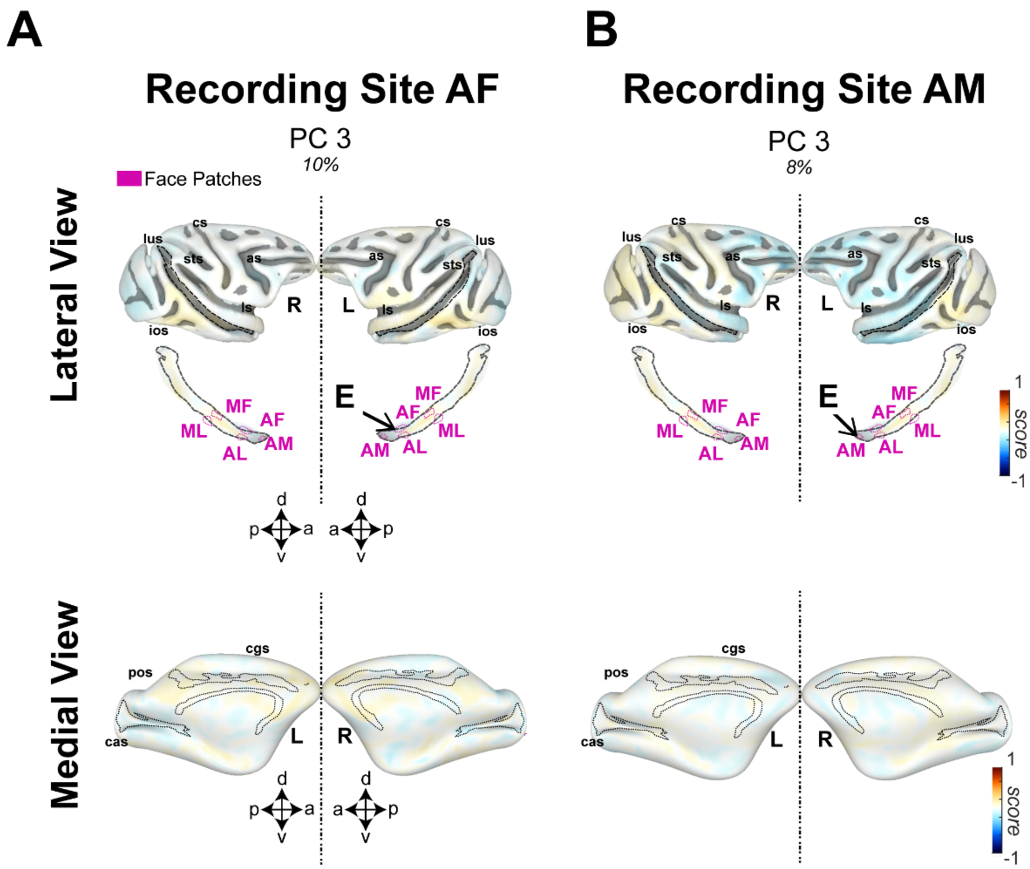

Fig. S7 Results from the third PC. Functional maps showing the results obtained from PCA analysis. Results are showing the third PC from recordings in **(A)** AF and in **(B)** AM. Lateral (top panel) and medial (bottom panel) views are shown for each corresponding recording site. “E” demarks the location of the electrode.

ROIs *(Abbreviations)*	Names *From Paxinos, Huan Atlas*
V1	Primary Visual Cortex
V1m	medial V1
V4	Area V4
V4v	Ventral V4
MT	
TEO	
F4	Frontal Area 4
F5	Frontal Area 5
Cla	Claustrum
Hip	Hippocampus
BL	Basolateral Amygdala
Str	Striatum
GP	Globus Pallidus
CeA	Central Amygdala
NBM	Nucleus Basalis Meynert
LGN	Lateral Geniculate Nucleus
MD	Medio Dorsal Thalamus
aPul	Anterior Pulvinar
iPul	Inferior Pulvinar
SN	Substantia Nigra
PPT	Pontinopedunculo Tegmental Area
iDR	Dorsal Raphe

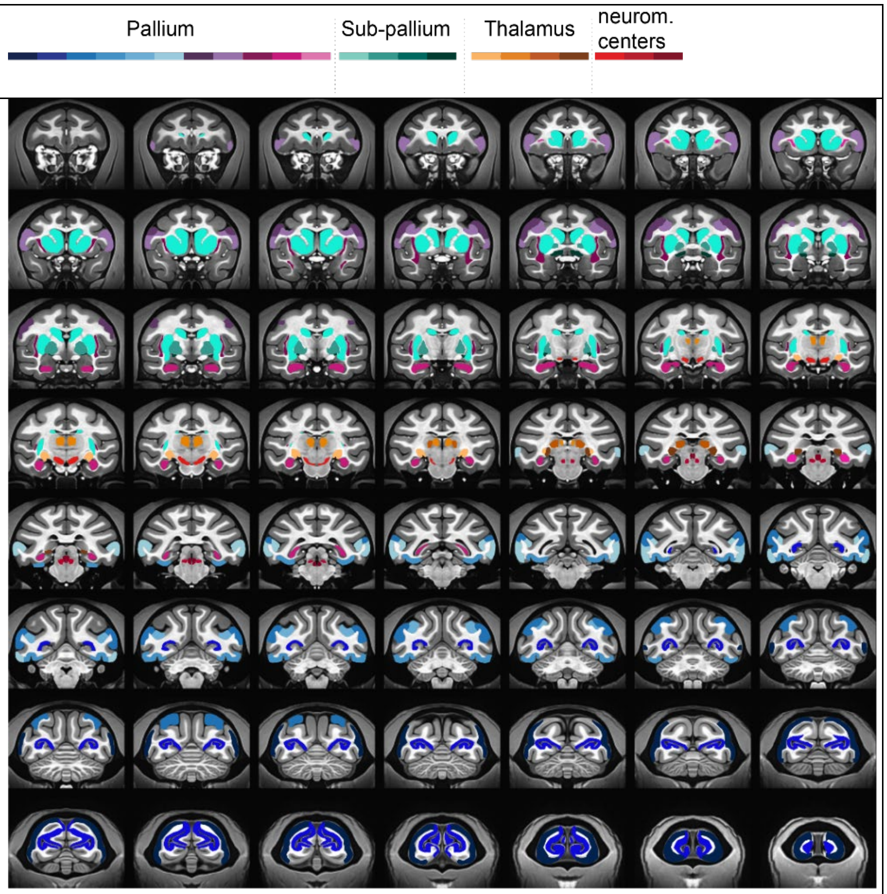

Fig. S8 Location and extent of ROIs. The color code and name list in the left indicate the ROIs' location in high-resolution MRI anatomical images. Cortical and subcortical structures are categorized according to their anatomical location relative to the subdivisions of the standard developmental brain (panel on top of anatomical images). The ROIs that we used here, as the initial template were first defined interactively using custom software on an average monkey brain.

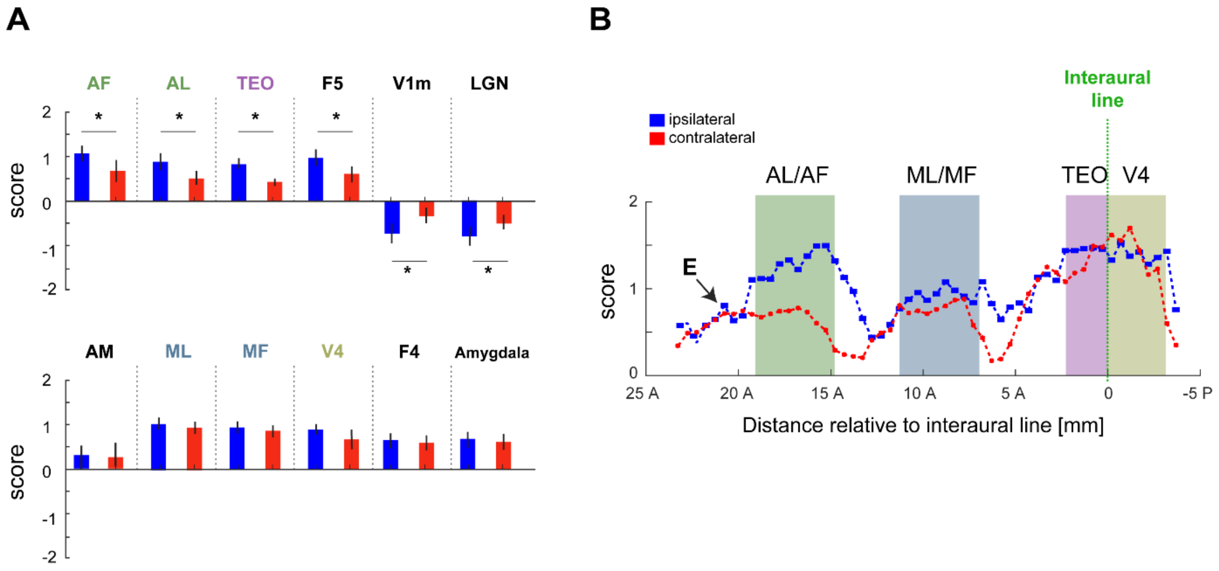

Fig. S9 Hemispheric differences in fMRI response in AM. Difference in functional activity between the recording hemisphere, or ipsilateral, and the contralateral hemisphere. **(A)** comparison between the ipsilateral (shown in blue) and the contralateral (shown in read) hemispheres from regions showing significant differences in their fMRI coupling to the spiking activity (top panel) and regions with no evident difference across the two hemispheres (bottom panel). **(B),** distribution of the fMRI coupling to the spiking activity along the V4/IT axis. The structure and organization in this figure are the same as in **Fig. 5**.

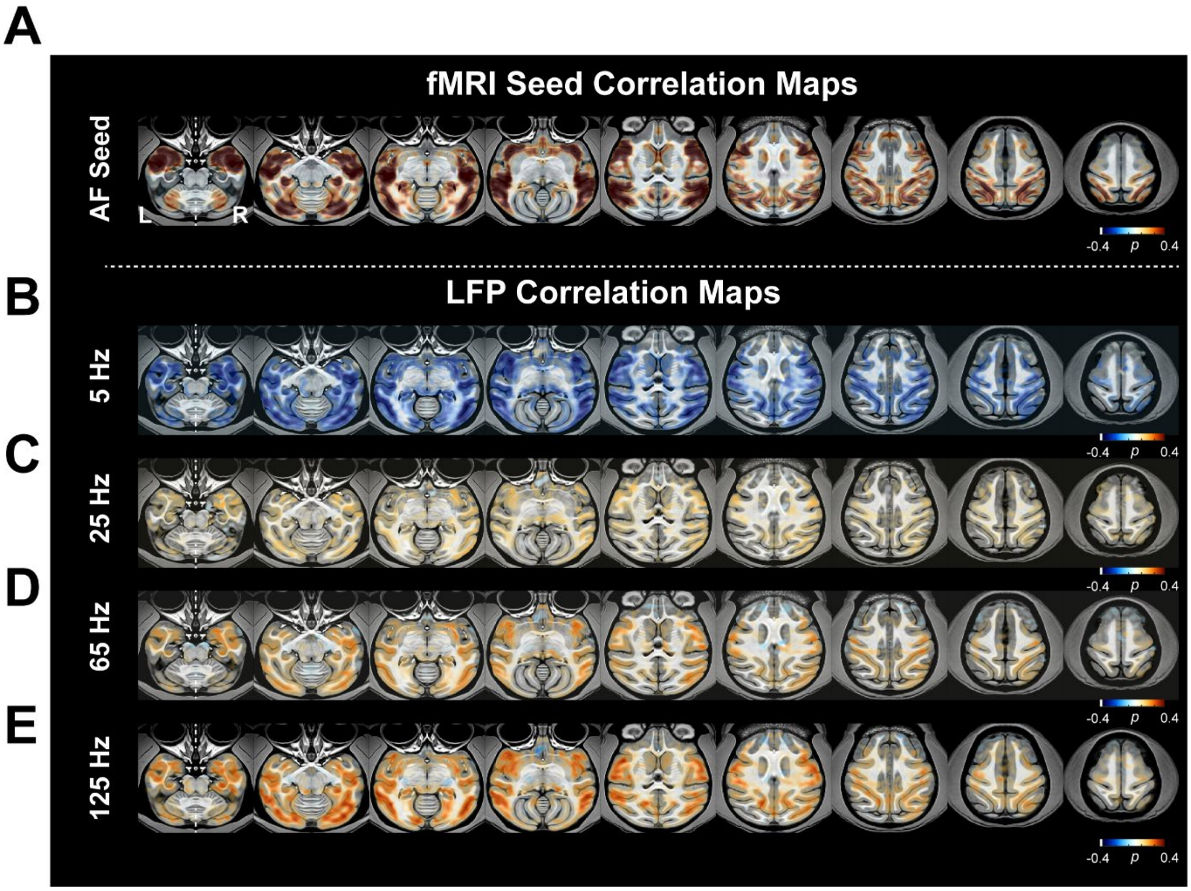

Fig. S10 Correlation maps based on the local population signals. Whole-brain functional maps extracted associated to the **(A)** fMRI seed in the AF face patch, and with selected LFPs bands: **(B)** 5 Hz; **(C)** 25 Hz; **(D)** 65 Hz; and **(E)** 125 Hz frequency ranges.

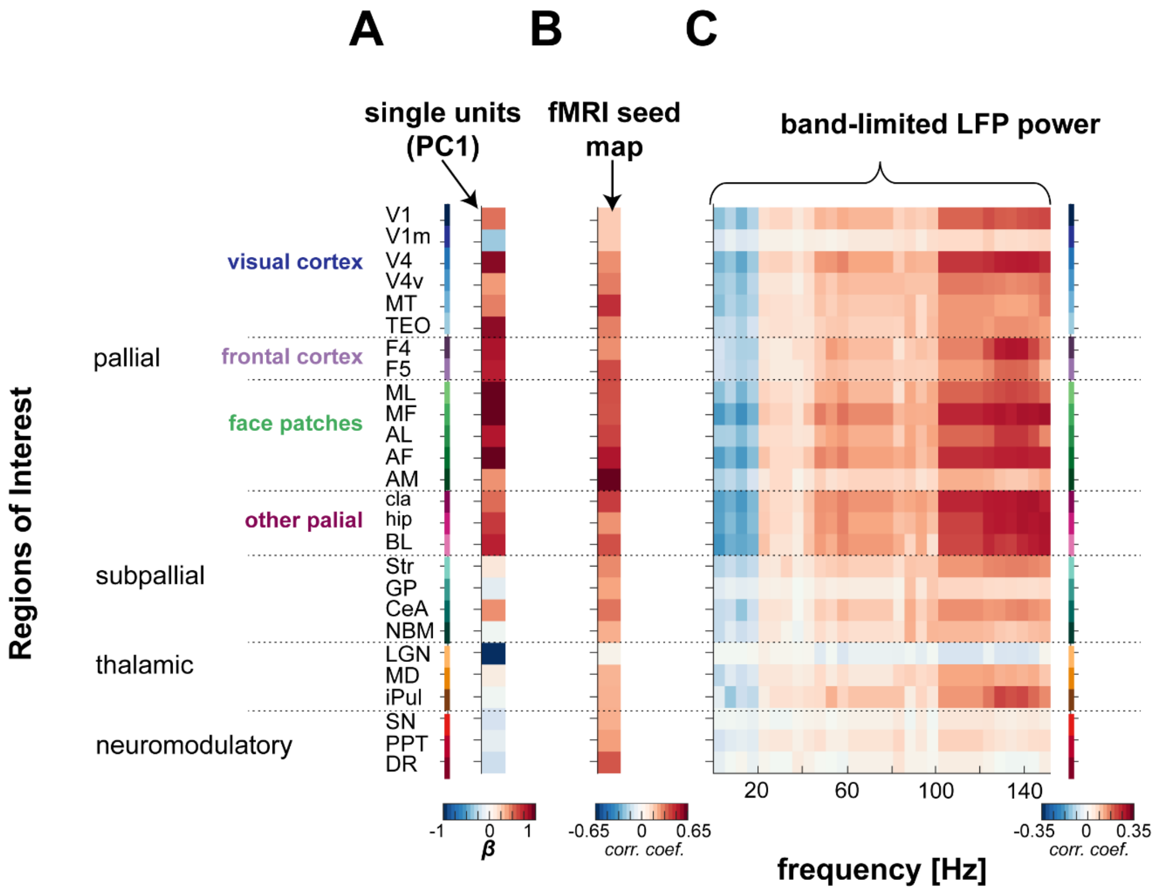

Fig. S11 Comparison of local population signals with the single-unit fMRI activity. **(A)** fMRI profiles from the population spiking activity in AM, **(B)** fMRI seed in AM (seed across n = 20 scans). **(C)** the correlation profiles across different LFP frequency bands spaced by 5 Hz each (n = 30 columns representing 30 LFP frequencies. The organization and structure in this figure are the same as in **Fig. 6** (see also **Fig. S8** for the ROIs names and their spatial extent).

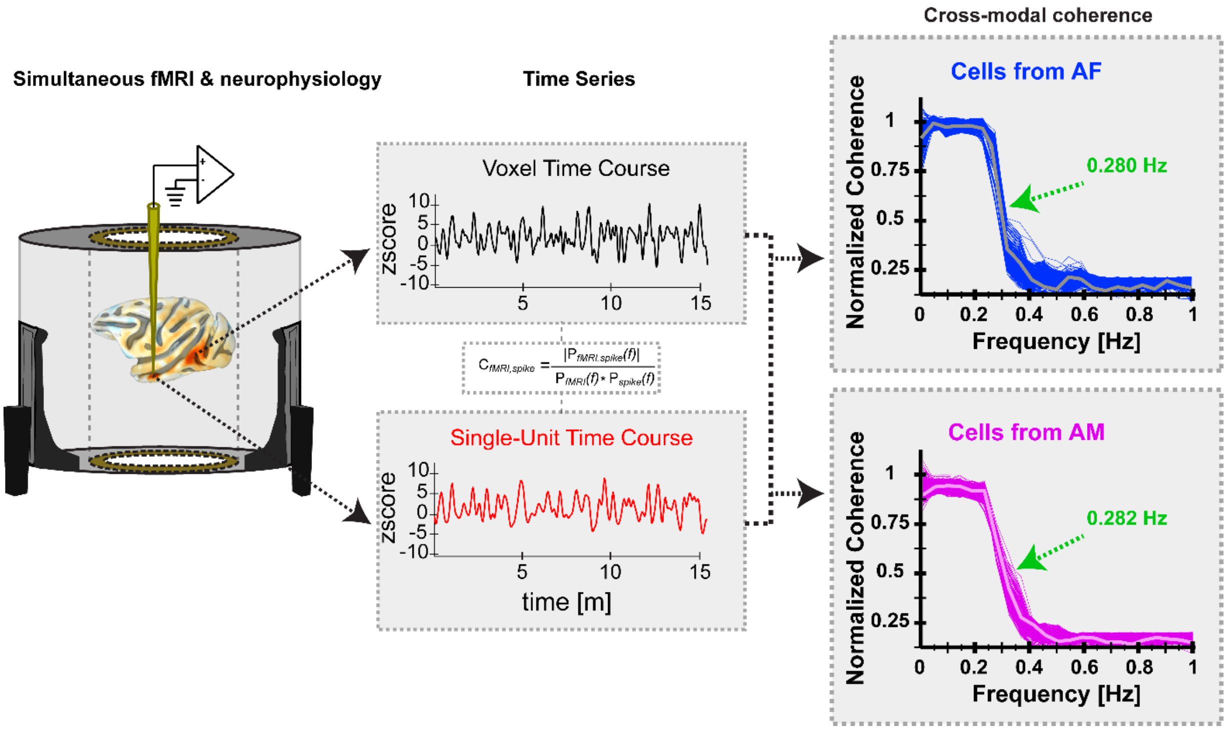

Fig. S12 Cross-modal relationship between fMRI and single unit spiking activity. We quantified the frequency correspondence between the spiking activity and fMRI voxels. Coherence estimates were computed as magnitude-squared coherence $C_{fMRI,spike}(f)$ using the Welch's average, modified periodogram method as shown in formula depicted in the figure. $P_{fMRI}(f)$ corresponds to the spectral densities from voxel's time courses in ROIs (n= 6, areas include face patches and V4) with high correlation values (total number of voxels = 852) and $P_{spike}(f)$ from time courses extracted from the spiking activity (in blue are 37 single units recorded from AF and in purple are 34 single units recorded from AM. The absolute coherence was normalized so that we could focus on the frequency cutoff which was estimated by taking the frequency at which coherence between the two signals dropped to half of its maximum value. For both AM and AF the cutoff was ~0.28 Hz (indicated by the green arrows in both AF and AM panels).
